# Does Real Time Ultrasonography Confer Any Benefit During Bronchoscopy Guided Percutaneous Tracheostomy: A Preliminary, Randomized Controlled Trial

**DOI:** 10.5005/jp-journals-10071-23169

**Published:** 2019-05

**Authors:** Richa Aggarwal, Kapil Dev Soni, Keshav Goyal, Gyaninder Pal Singh, Navdeep Sokhal, Anjan Trikha

**Affiliations:** 1,2 Department of Critical and Intensive Care, JPNATC, All India Institute of Medical Sciences, New Delhi, India; 3-5 Department of Neuroanaesthesia and Critical Care, AIIMS, New Delhi, India; 6 Department of Anaesthesia, Critical Care and Pain Medicine, AIIMS, New Delhi, India

**Keywords:** Bronchoscopy, Percutaneous dilatational tracheostomy, Ultrasonography

## Abstract

**Background:**

There are studies comparing USG guided percutaneous dilatational tracheostomy (PDT) with bronchoscopy guided PDT. We have compared USG guided PDT to conventional landmark guided PDT using bronchoscopy in both the groups.

**Objective:**

The primary outcome was the time of procedure and the secondary outcome was incidence of complications in USG guided PDT in comparision to the conventional PDT.

**Materials and Methods:**

Seventy adult patients were randomly allocated in two groups, i.e., conventional landmark percutaneous dilatational tracheostomy (PDT) and ultrasonography (USG) guided PDT. Demographic data, injury severity score, time taken for the procedure, attempts of tracheal puncture, major and minor complications, and outcome were compared.

**Results:**

The median time taken for the procedure was 12 minutes [min., max.; 8, 20] in conventional group 1 and 16 minutes [9, 24] in group 2 (USG guided) which was statistically significant. Minor bleeding was seen in 7 (20%) patients in group 1 and only in 4 patients (11.5%) in group 2. The rate of other complications and the long term outcome were similar in both the groups.

**Conclusion:**

The use of real time USG during PDT may confer advantage over conventional PDT when using bronchoscopy in terms of decreasing the incidence of minor bleeding but duration of the procedure gets prolonged.

**How to cite this article:**

Aggarwal R, Soni KD, Goyal K, Singh GP, Sokhal N, Trikha A. Does Real Time Ultrasonography Confer Any Benefit During Bronchoscopy Guided Percutaneous Tracheostomy: A Preliminary, Randomized Controlled Trial. Indian J Crit Care Med 2019;23(5):236–238.

## INTRODUCTION

Percutaneous dilatational tracheostomy (PDT) has almost replaced surgical tracheostomy in intensive care unit (ICU) patients as it is simpler, safer, and less time consuming bedside procedure. Furthermore, bronchoscopy is being used commonly to guide PDT in order to decrease complications.^1^ To make it more safe, real time ultrasonography (USG) is being used now a days to guide needle insertion during PDT.^2–4^ Few controlled trials have proven USG guided PDT as noninferior, easier, and faster than bronchoscopy guided PDT with less complications.^5,6^ However, it is not clear whether real time ultrasonography confer any benefit during bronchoscopy guided percutaneous tracheostomy. We conducted a randomized controlled trial to compare real time USG guided PDT with conventional PDT while using bronchoscopy in both the groups.

## MATERIALS AND METHODS

The study was a prospective randomized controlled trial done in surgical ICU of level-1 trauma centre in India. The primary outcome was the time of procedure in two groups which was defined as the time after cleaning and draping the patient till the confirmation of tube by bronchoscopy. The secondary aim was to find the difference in the ease of procedure, the attempts taken for tracheal puncture, the rate of complications, and the clinical outcomes. Major complications included major bleeding (requiring surgical control or blood transfusion), hypoxemia (oxygen peripheral saturation below 90 % for more than 5 minutes), persistent hypotension (systolic blood pressure below 90 mm Hg for more than 5 minutes during the procedure), posterior tracheal wall rupture, malposition of the tracheostomy tube or pneumothorax, while others were minor complications. Clinical outcomes were time taken from tracheostomy to unassisted breathing, ICU mortality, and hospital length of stay.

Adult patients age >18 years were considered as eligible. Patients with short neck, who had a tracheostomy/any neck surgery in the past or with tracheal injury were excluded.

After approval from ethics committee and informed consent, 70 adult patients were enrolled. Patients were randomly allocated in two groups of 35 each in 1:1 ratio. A standard randomization procedure for allocation in each group was followed using nQuery 2.0 software.

**Fig. 1:**
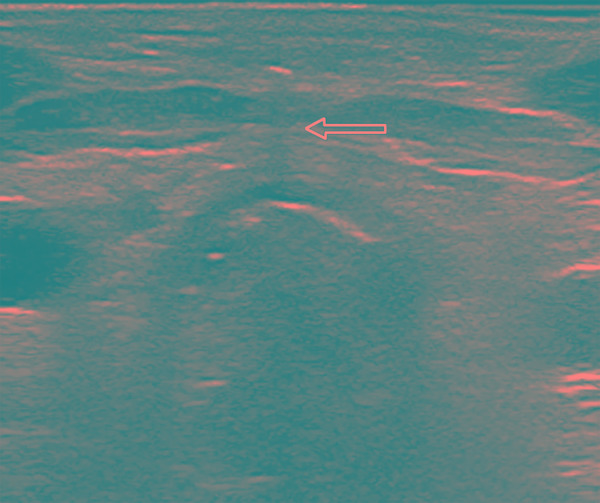
Transverse view of trachea showing the acoustic shadow (arrow) depicting the path of the needle used for tracheal puncture

The PDT was performed in both the groups using the single step, progressive Ciaglia Blue Rhino technique^7^ using the Ciaglia Blue Rhino kit. In the patients randomized to the USG group, tracheal puncture was performed under USG guidance whereas in conventional group, tracheal puncture was performed blindly using traditional landmarks. Tracheal puncture was done using a transverse probe position and the needle was visualized in an out of plane mode (Fig. 1). The video bronchoscope was then passed through the endotracheal tube, the exact point of needle entry was recorded, and the trachea was visualized for any signs of injury or posterior wall puncture. The guidewire was introduced under the vision of the bronchoscope. In the USG group, the guide wire was visualized as hyperechoic signal on transverse and longitudinal sections. The dilatation of the tract was done by an initial small dilator and then Ciaglia Blue Rhino and tracheostomy tube was placed. Position of the tube was confirmed by auscultation and bronchoscopy.

The procedure was done by either of the two intensive care consultants and not by residents to decrease the subjective differences.

### Statistical Analysis

As per the pilot study done in 10 patients, assuming that the time taken with USG is 25±7 minutes and without USG 19±8 minutes with alpha 5% and power 90%, 66 cases were required, 33 in each group. We enrolled 35 patients in each group. For comparison, chi square test was applied for qualitative data and student's t test or Mann Whitney test for quantitative data analysis.

## RESULTS

Seventy patients were prospectively enrolled in the study between January 2014 to February 2016 with 35 patients in each group. Demographic data and baseline characteristics like injury severity score and ventilator days prior to tracheostomy were similar in both the groups.

Similar number of attempts were required in both the groups for tracheal puncture. The median time taken for the procedure was 12 minutes [min., max.; 8, 20] in conventional group 1 and 16 minutes [9, 24] in group 2 which was statistically significant. The procedure difficulty was similar in both groups. It was graded as easy in 62% patients in group 1 and in 68% in group 2. No patient required conversion to surgical tracheostomy (Table 1).

### Complications and Outcome

Minor bleeding was seen in 7 (20%) patients in group 1 and only in 4 patients (11.5%) in group 2 while major bleeding was infrequent. The incidence of other complications were similar in both the groups. Local infection of the tracheostomy site was seen more in group 2 (28.5% vs 17.1%) than group 1. The median time from tracheostomy to unassisted breathing was similar in two groups—12 days in group 1 and 8 days in group 2. There was no difference in the hospital length of stay and ICU mortality in two groups (Table 1).

## DISCUSSION

Fibreoptic bronchoscopy is being used commonly to guide PDT in order to decrease complications like puncture of the posterior tracheal wall and formation of false passage.^1^ However, bronchoscopy does not help in controlling hemorrhage and pointing the exact site of needle insertion. For this, pre-procedure USG of the neck^8^ and real time USG has been advocated. Rajajee et al.^2^ and Chacko et al.^4^ used real time USG to guide PDT and conducted feasibility studies. Real time USG not only guides the needle placement below the first tracheal ring but also allows the visual confirmation that the anterior wall of the trachea has been passed and this may avoid injury to the posterior tracheal wall.

However, the concern regarding real time USG is that direct visualization of the needle is not possible in all patients^9^ and USG cannot be used to rule out injury to the posterior wall of the trachea. Bronchoscopy has the advantage like greater control of airway, better visualization of the entire procedure and the risk of puncture to the posterior wall of the trachea is reduced.

Two trials have compared USG guided tracheostomy with bronchoscopy guided tracheostomy.^5,6^ However, Yavuz et al.^10^ and Rudas et al.^11^ have compared traditional landmark with USG guided tracheostomy but only Rudas et al.^11^ have used bronchoscopy in both the groups. We have compared USG guided tracheostomy to landmark guided tracheostomy using bronchoscopy in both the groups as bronchoscopy guided tracheostomy is the standard method of tracheostomy in our ICU for the past 8 years.

Rudas et al.^11^ showed higher first pass success rate of 87% vs 58% in patients with USG. Similarly, Yavuz et al.^10^ showed that patients requiring multiple attempts were fewer with USG (4% vs 14%). In our study also, first pass rate was higher in USG group (68% vs 62%) but was not statistically significant.

Ease of procedure was graded similar for both the groups in our study which was also reported by earlier studies. We had excluded patients with difficult anatomy. Time taken for USG group was more in our study as compared to control group (12 vs 16 minutes) and this was statistically significant (*p* <0.001). Yuvaz et al.^10^, similarly had prolonged duration of procedure (24 vs 19 minutes) in USG group. However, in study by Ravi and Vijay^5^, the time taken for tracheostomy was significantly lower in the USG group (16 vs 12 minutes). The reason for this could be that they measured the time between the puncture of the trachea and the ventilation of the patient whereas in our study, the time after draping the patient till the confirmation of the tube by bronchoscopy was measured so more time was spent in USG neck examination.

The incidence of overall complications like hypotension, transient desaturation, and needle hitting the endotracheal tube were similar in both the groups. Ravi and Vijay^5^ experienced increased incidence of hypoxia in bronchoscopy group than USG group and hypothesized that hypoxia could be due to breach in the continuity of the close airway for the insertion of the bronchoscope. We did not find difference in the groups as we used bronchoscopy in both the groups and used the connector through which we could ventilate the patient while doing bronchoscopy.

**Table 1:** Comparison between the two groups, demographic variables, procedure, complications and outcome

*Variables*	*Gp 1 (without USG)*	*Gp 2 (with USG)*	*p value*
Age (yrs)*	33 [12.7]	34 [11.8]	0.7866
M:F	31:4	34: 1	
ISS*	19.1 [8.6]	18.3 [8.1]	0.6716
VD before tracheostomy	12 [3, 20]	14 [4, 20]	0.1999
Time taken for tracheostomy	12 [8, 20]	16 [9, 24]	<0.001
Procedure difficulty (%)			0.734
Easy	62	68	
Somewhat difficult	31	22	
Very difficult	5	8	
Tracheal punctures (%)			0.611
1st attempt	62	68	
2nd attempt	37	28	
3rd attempt	0	0.28	
Surgical conversion complications, n (%)	0	0	1.000
Transient hypotension, *n* (%)	6 [17.1]	4 [11.4]	0.734
Transient hypoxia	8 [22.8]	7 [20]	0.771
Minor bleeding	7 [20]	4 [11.5]	0.203
Tracheal cuff puncture	0 [0]	0 [0]	
Needle hitting the endotracheal tube	8 [22.8]	8 [22.8]	1.000
Infection at the stoma site	6 [17.14]	10 [28.5]	0.255
Length of hospital stay	31 [10, 290]	32 [13, 210]	0.8125
Mortality	26 [74.29]	26 [74.29]	1.000
Time to wean off the ventilator	12 [0, 44]	8 [0, 65]	0.8879

*Gp*: Group; *VD*: Ventilator days; *ISS*: Injury severity score

Values are expressed as the *mean (standard deviation), median [min., max.], or a number (percentage)

The incidence of minor bleeding was less in USG group (20% vs 11.5%) though statistically insignificant, which is in accordance with other studies. However, the incidence of infection was more in the USG group (17% vs 28.5%). Time taken from tracheostomy to unassisted breathing, hospital length of stay, and mortality was similar in both the groups as reported in literature.

## CONCLUSION

The use of real time USG during PDT confer advantage over conventional PDT when using bronchoscopy in terms of decreasing the incidence of minor bleeding and may increase the first attempt pass rate of the needle (though not statistically significant) but prolongs the time of the procedure. Bronchoscopy and USG are not totally comparative and should be used in combination to improve the PDT safety and efficacy.

## References

[1] Tomsic JP,, Connolly MC,, Joe VC,, Wong DT. (2006;). Evaluation of bronchoscopic-assisted percutaneous tracheostomy.. Am Surg..

[2] Rajajee V,, Fletcher JJ,, Rochlen LR,, Jacobs TL. (2011;). Real-time ultrasound-guided percutaneous dilatational tracheostomy: a feasibility study.. Crit Care..

[3] Guinot P,, Zogheib E,, Petiot S,, Marienne JP,, Guerin AM,, Monet P, (2012;). Ultrasound-guided percutaneous tracheostomy in critically ill obese patients.. Crit Care..

[4] Chacko J,, Nikahat J,, Gagan B,, Umesh K,, Ramanathan M. (2012;). Real-time ultrasound-guided percutaneous dilatational tracheostomy.. Intensive Care Med..

[5] Ravi PR,, Vijay MN. (2015;). Real time ultrasound-guided percutaneous tracheostomy: Is it a better option than bronchoscopic guided percutaneous tracheostomy?. Med J Armed Forces India..

[6] Gobatto ALN,, Besen BAMP,, Tierno PFGMM,, Mendes PV,, Cadamuro F,, Joelsons D, (2016;). Ultrasound-guided percutaneous dilational tracheostomy versus bronchoscopy-guided percutaneous dilational tracheostomy in critically ill patients (TRACHUS): a randomized noninferiority controlled trial.. Intensive Care Med..

[7] Byhahn C,, Wilke HJ,, Halbig S,, Lischke V,, Westphal K. (2000;). Percutaneous tracheostomy: Ciaglia Blue Rhino versus the basic Ciaglia technique of percutaneous dilational tracheostomy.. Anesth Analg..

[8] Hartfield A,, Bodenham A. (1999;). Portable ultrasonic scanning of the anterior neck before percutaneous dilatational tracheostomy.. Anaesthesia..

[9] Tremblay LN,, Scales DC. (2011;). Ultrasound-guided tracheostomy–not for the many, but perhaps the few… or the one.. Crit Care..

[10] Yavuz A,, Yilmaz M,, Goya C,, Alimoglu E,, Kabaalioglu A. (2014;). Advantages of US in percutaneous dilatational tracheostomy: randomized controlled trial and review of the literature.. Radiology..

[11] Rudas M,, Seppelt I,, Herkes R,, Hislop R,, Rajbhandari D,, Weisbrodt L. (2014;). Traditional landmark versus ultrasound guided tracheal puncture during percutaneous dilatational tracheostomy in adult intensive care patients: a randomised controlled trial.. Crit Care..

